# Use of Third Line Antiretroviral Therapy in Latin America

**DOI:** 10.1371/journal.pone.0106887

**Published:** 2014-09-15

**Authors:** Carina Cesar, Bryan E. Shepherd, Cathy A. Jenkins, Massimo Ghidinelli, Jose Luis Castro, Valdiléa Gonçalves Veloso, Claudia P. Cortes, Denis Padgett, Brenda Crabtree-Ramirez, Eduardo Gotuzzo, Valeria Fink, Adriana Duran, Omar Sued, Catherine C. McGowan, Pedro Cahn, for The Caribbean, Central and South America Network for HIV Epidemiology (CCASAnet)

**Affiliations:** 1 Fundación Huésped, Buenos Aires, Argentina; 2 Vanderbilt University, Nashville, Tennessee, United States of America; 3 Pan American Health Organization, Washington, D.C., United States of America; 4 Instituto de Pesquisa Clinica Evandro Chagas-Fundação Oswaldo Cruz, Rio de Janeiro, Brazil; 5 Universidad de Chile, Santiago, Chile; 6 Instituto Hondureño de Seguridad Social and Hospital Escuela, Tegucigalpa, Honduras; 7 Instituto Nacional de Ciencias Médicas y Nutrición, Mexico City, Mexico; 8 Instituto de Medicina Tropical Alexander von Humboldt, Lima, Peru; 9 Coordinación Sida, Ministerio de Salud de la Ciudad Autónoma de Buenos Aires, Buenos Aires, Argentina; McGill University AIDS Centre, Canada

## Abstract

**Background:**

Access to highly active antiretroviral therapy (HAART) is expanding in Latin America. Many patients require second and third line therapy due to toxicity, tolerability, failure, or a combination of factors. The need for third line HAART, essential for program planning, is not known.

**Methods:**

Antiretroviral-naïve patients ≥18 years who started first HAART after January 1, 2000 in Caribbean, Central and South America Network (CCASAnet) sites in Argentina, Brazil, Honduras, Mexico, and Peru were included. Clinical trials participants were excluded. Third line HAART was defined as use of darunavir, tipranavir, etravirine, enfuvirtide, maraviroc or raltegravir. Need for third line HAART was defined as virologic failure while on second line HAART.

**Results:**

Of 5853 HAART initiators followed for a median of 3.5 years, 310 (5.3%) failed a second line regimen and 44 (0.8%) received a third line regimen. Cumulative incidence of failing a 2nd or starting a 3rd line regimen was 2.7% and 6.0% three and five years after HAART initiation, respectively. Predictors at HAART initiation for failing a second or starting a third line included female sex (hazard ratio [HR] = 1.54, 95% confidence interval [CI] 1.18–2.00, p = 0.001), younger age (HR = 2.76 for 20 vs. 40 years, 95% CI 1.86–4.10, p<0.001), and prior AIDS (HR = 2.17, 95% CI 1.62–2.90, p<0.001).

**Conclusions:**

Third line regimens may be needed for at least 6% of patients in Latin America within 5 years of starting HAART, a substantial proportion given the large numbers of patients on HAART in the region. Improved accessibility to third line regimens is warranted.

## Introduction

Unprecedented global efforts have resulted in the rapid expansion of access to highly active antiretroviral therapy (HAART) throughout the world. In 2002 only 300,000 people living with HIV were receiving HAART in low- and middle-income countries; in 2012 this figure increased to 9.7 million [1], [2]. Access to HAART is considered the primary means to increase life expectancy among the HIV-infected population and to reduce transmission to HIV-negative individuals. As the number of persons initiating first-line HAART increases worldwide, the number of people in need for both second- and third-line regimens will also increase.

Compared to other resource-limited settings (RLS), Latin America and the Caribbean (LAC) have had higher rates of HAART coverage. In 2012, 715,000 people were receiving HAART in low and middle income countries within LAC, accounting for a regional coverage of more than 70% [1], [2]. Many South American countries started to provide HAART through public health systems early in the 1990's. By 2002, several countries had boosted their national HIV/AIDS budgets, while Central American and Caribbean countries had seen an increase in external resources for AIDS [3]. As HIV/AIDS treatment programs have expanded in RLS, an increasing number of people living with HIV have needed second-line regimens. In LAC, the percentage of individuals receiving second-line regimens is higher than that reported in other RLS: 27% of patients (ranging from 4 to 43%) are receiving second-line regimens compared with 0.05% in other regions of the developing world [2], [3]. This may in part be due to specific characteristics of the Americas region, such as the age of national programs, with many patients starting HAART before 2000, the use of individualized approaches for treating HIV-infected persons, access to broader HAART options, and an increasing frequency of viral load determinations. According to a public health analysis of HAART in the Caribbean and Latin America, viral load determinations increased from a median 1.2 per year in 2010 to 1.8 in 2012 [4].

At a global level, increased attention is being paid towards second line HAART regimens, particularly their outcomes and associated factors [5]–[10]. As the number of patients receiving second line HAART is increasing, the need for third line regimens is also likely to increase in the next few years. Although from a logical standpoint, rational and efficient use of resources, good patient adherence to HAART, and high quality care would reduce the need for third-line drugs, there are no published data on factors associated with the need for third-line drugs in the LAC region.

Therefore, the objectives of this study were to estimate the current need and use of third line regimens among patients initiating antiretroviral therapy in Latin America and to determine factors associated with need for third line drugs.

## Methods

The Caribbean, Central and South America Network (CCASAnet) has been described elsewhere [11]. Briefly, CCASAnet is a consortium of HIV clinics from 7 countries (Argentina, Brazil, Chile, Haiti, Honduras, Mexico and Peru), within the International epidemiologic Databases to Evaluate AIDS (IeDEA). Sites contributing data to this study were the following: Centro Médico Huésped, Buenos Aires, Argentina (CMH-Argentina); Instituto de Pesquisa Clinica Evandro Chagas, Fundação Oswaldo Cruz, Rio de Janeiro, Brazil (FC- Brazil); Instituto Hondureño de Seguridad Social and Hospital Escuela, Tegucigalpa, Honduras (IHSS/HE-Honduras); Instituto Nacional de Ciencias Médicas y Nutrición Salvador Zubirán, Mexico City, Mexico (INCMNSZ-Mexico); and Instituto de Medicina Tropical Alexander von Humboldt, Lima, Perú (IMTAvH-Peru). Clinical data were collected at each site, de-identified, and sent to the CCASAnet Data Coordinating Center at Vanderbilt University, Nashville, TN, USA (VDCC), for data harmonization and processing. The VDCC checked data for internal consistency and performed periodic quality assessment of data collection and validation through on-site data audits. Institutional ethics review boards from all sites and Vanderbilt University reviewed and approved the project. Antiretroviral-naïve adults (≥18 years) initiating their first HAART regimen at participating CCASAnet sites on or after January 1, 2000 were included in this study. Database closing varied by site; latest dates of HAART initiation were October 2012 (CMH-Argentina), April 2013 (FH-Brazil), February 2013 (IHSS/HE-Honduras and INCMNSZ-Mexico), and May 2013 (IMTAvH-Peru). Patients known to be participating in any clinical intervention protocol were excluded from analyses.

The primary outcomes were: 1) starting a third line regimen and 2) virologic failure after starting a second line regimen. A third line regimen was defined as any regimen containing one of the following drugs: darunavir, tipranavir, etravirine, enfuvirtide, maraviroc or raltegravir. A second line regimen was defined as a regimen containing a boosted protease inhibitor (PI) preceded by a regimen containing either a different PI or a non-nucleoside reverse transcriptase inhibitor (NNRTI). Virologic failure after starting a second line regimen was defined as one of the following: 1) HIV-1 RNA level never dropped below 400 copies/ml after 6 months of therapy, 2) HIV-1 RNA level dropped below 400 copies/ml but then there were two consecutive values>400 copies/ml (without regards to time between measurements), 3) HIV-1 RNA level dropped below 400 copies/ml but then there was a single measurement>1000 copies/ml. The sensitivity of the results to this definition of virologic failure was investigated by removing the third criterion. The cutoff of 400 copies/ml was chosen because it was the HIV-1 RNA detection limit for many of the sites over much of the study period.

Clinical stage prior to initiation of first HAART was categorized as AIDS or not AIDS; clinical AIDS was defined as CDC stage C, WHO stage IV, or a specification of AIDS at first visit. CD4 at HAART initiation was defined as the CD4 cell count closest to HAART initiation but no more than 180 days prior to or 7 days after. HIV-1 RNA at HAART initiation was defined as the measurement closest to initiating HAART but no more than 180 days before; any HIV-1 RNA measurement after initiating was not included. HIV-1 RNA at the start of the second line regimen was similarly defined.

The time until either of the two outcomes (starting a third line regimen or virologic failure while on a second line regimen) was assessed as an approximate measure of the time until the need for third line HAART. The cumulative incidence after HAART initiation (time 0) of the composite endpoint was estimated treating death as a competing event. The cumulative incidence of loss to follow-up, defined as no patient laboratory or medical visit within the year prior to the database closing date, was also estimated treating death as a competing event. Risk factors for the composite endpoint were assessed using Cox proportional hazards models. All models (both unadjusted and adjusted) were stratified by site. The primary adjusted models included sex, probable route of infection (heterosexual, men who have sex with men [MSM], injection drug use [IDU], or other), and calendar year, age, CD4 count, and AIDS at HAART initiation. Covariates were chosen a priori based on availability and perceived relevance. Secondary analyses also included pre-cHAART log-transformed HIV-1 RNA levels. The adjusted analyses used multiple imputation to account for missing data. Age, pre-HAART CD4 count, calendar year, and log-transformed HIV-1 RNA levels were included in the models using restricted cubic splines with 3 knots to relax linearity assumptions. The proportional hazards assumption was assessed using a global test for correlation between scaled Schoenfeld residuals and time; proportional hazards was deemed reasonable (p>0.05). All analyses were performed using R Statistical Software; analysis scripts are available at http://biostat.mc.vanderbilt.edu/ArchivedAnalyses.

## Results

A total of 5853 HAART initiating patients were included in this study: 882 from CMH-Argentina, 1382 from FC-Brazil, 918 from IHSS/HE-Honduras, 674 from INCMNSZ-Mexico, and 1997 from IMTAvH-Peru. Patient characteristics at HAART initiation are shown in Table 1. Median age was 35 years, males accounted for 70% of patients, median CD4 was 147 cells/mm^3^, and 41% had AIDS prior to HAART initiation. The vast majority of patients (85%) started a regimen containing an NNRTI; boosted PIs were prescribed as the first regimen for 697patients (12%). The median follow-up was 3.5 years (interquartile range [IQR] 1.5–6.0). The cumulative incidence of loss to follow-up was 8% at 3 years, 14% at 5 years, and 21% at 7 years. The average frequency of HIV-1 RNA measurements recorded in the database was 2.6 per year, although this varied by site (ranging from a median of 0.8 measurements per year in IHSS/HE-Honduras to a median of 3.4 in CMH-Argentina).

**Table 1:** Cohort description.

	N	Summary
Male	5853	70% (4078)
Age of first HAART	5853	35 (29–43)
Probable route of infection	5853	
Heterosexual		51% (2986)
MSM		30% (1783)
IDU		1% (38)
Other/Unknown		18% (1046)
AIDS at first HAART	4776	41% (1953)
CD4 at first HAART	5032	147 (56–259)
HIV-1 RNA at first HAART (log_10_)	3798	5.0 (4.5–5.4)
Initial HAART regimen	5853	
NNRTI		85% (4988)
BOOSTED PI		12% (697)
PI		2% (120)
3 NRTI		1% (44)
Other		0% (4)
Site	5853	
CMH-Argentina		15% (882)
FC-Brazil		24% (1382)
IHSS/HE-Honduras		16% (918)
INCMNSZ-Mexico		12% (674)
IMTAvH-Peru		34% (1997)
Years of Follow-up	5853	3.5 (1.5–6.0)

Categorical variables are reported as percentages (count).

Continuous variables are reported as medians (interquartile range).

Over the course of follow-up, 993 patients (17%) started a second line regimen. Of these patients, 310 (31% of those starting a second line) experienced virologic failure after starting the second line regimen. Characteristics of these patients are shown in Table 2. The median time from HAART initiation to second line failure was 3.3 years (IQR 1.9 to 5.0 years). These patients were highly immunocompromised at HAART initiation with a median CD4 of 107 cells/mm^3^ (IQR 38 to 246) and 62% experienced a prior AIDS event. Thirty-six (12%) of these patients subsequently died. Of note, 32 (10%) of these failures occurred while there was a reported treatment interruption. In secondary analyses defining virologic failure as two consecutive HIV-1 RNA measurements>400 copies/ml, 174 (18% of those starting a second line or 3% of HAART initiators) experienced virologic failure after starting a second line regimen.

**Table 2:** Second line virologic failure. Patient descriptive statistics.

	N	Summary
Age at first HAART	310	33 (28–40)
Male	310	62% (191)
Probable route of infection	310	
Heterosexual		52% (162)
MSM		27% (83)
IDU		2% (6)
Other/Unknown		19% (59)
AIDS at first HAART	252	62% (155)
CD4 at first HAART	244	107 (38–246)
HIV-1 RNA at first HAART (log_10_)	198	5.0 (4.6–5.5)
Initial HAART	310	
NNRTI		80% (249)
BOOSTED PI		15% (47)
PI		3% (8)
3 NRTI		2% (6)
Other		0% (0)
Site	310	
CMH-Argentina		18% (57)
FC-Brazil		40% (124)
IHSS/HE-Honduras		5% (15)
INCMNSZ-Mexico		10% (31)
IMTAvH-Peru		27% (83)
Started a 3rd line regimen	310	
Yes		7% (21)
Years from HAART start to 2nd line failure	310	3.3 (1.9–5.0)
Years from start of 2nd line regimen to failure	310	0.9 (0.5–1.7)
Second line regimen	310	
3TC, AZT, LPV, RTV		20% (62)
3TC, DDI, LPV, RTV		9% (27)
3TC, D4T, LPV, RTV		8% (24)
3TC, ATV, AZT, RTV		7% (22)
3TC, ATV, RTV, TDF		6% (20)
OTHER		50% (155)

Categorical variables are reported as percentages (count).

Continuous variables are reported as medians (interquartile range).

Over the entire follow-up period, 44 (0.8%) patients started a third line regimen. Among the 44 third line regimens started, 37 (84%) contained raltegravir, 20 (45%) contained darunavir, 10 (23%) contained etravirine, and 1 (2%) contained enfuvirtide. More common third-line regimens were: raltegravir + boosted PI not darunavir (22, 38%), raltegravir + darunavir (12, 21%), darunavir + NRTIs (10, 17%) and raltegravir + NRTIs (6, 10%). Characteristics of patients starting a third line regimen are shown in Table 3. The number of regimens prior to starting the third line regimen ranged from 1 to 8, with a median of 3 prior regimens. Eight patients had only one prior regimen, and therefore changed directly from first to third line. (Six of these patients started with an NNRTI-based regimen and two started with a boosted PI; three were reported to have changed to a third line due to virologic failure, the other five changed for other unspecified reasons.) While the median time from initiating HAART until failing second line therapy was 3.3 years, the median time from initiating HAART until starting a third line regimen was 4.0 years (IQR 1.6 to 7.6). For those who started a second line regimen prior to starting a third line regimen, the median time from initiating the second line regimen to starting a third line regimen was 2.4 years (IQR 1.5 to 3.4). Again, patients starting a third line regimen were highly immunocompromised at HAART initiation, with a median CD4 of 63 cells/mm^3^ (IQR 26 to 130) and 26 (63%) having had prior AIDS. Notably, no patient from IMTAvH-Peru and only one from IHSS/HE-Honduras started a third line regimen. Five (11%) patients starting third line therapy subsequently died; deaths occurred 0.5, 3.2, 3.2, 4.8, and 6.3 months after starting the third line regimen. Of those who failed a second line regimen who did not start a third line regimen or die (n = 256), 51% (n = 131) changed to at least one additional subsequent regimen during follow-up, but none of these regimens met the definition for third line. Of the 68 who had only 1 subsequent regimen, 61 experienced virologic failure, 4 re-suppressed and 3 did not have viral load determinations.

**Table 3:** Third line regimen patient's characteristics.

	N	Summary
Age at first HAART	44	34 (31–44)
Male	44	68% (30)
Probable route of infection	44	
Heterosexual		41% (18)
MSM		39% (17)
IDU		0% (0)
Other/Unknown		20% (9)
AIDS at first HAART	41	63% (26)
CD4 at first HAART	34	63 (26–130)
CD4 closest to 3rd line regimen	37	165 (83–348)
HIV1-RNA at first HAART (log_10_)	24	4.9 (4.4–5.0)
HIV1-RNA closest to 3rd line regimen	38	3.7 (2.9–4.4)
Years from HAART start to 3rd line regimen	44	4.0 (1.6–7.6)
Number of regimens prior to 3rd line	44	
1		18% (8)
2		25% (11)
3		20% (9)
4		23% (10)
5		5% (2)
6		5% (2)
8		5% (2)
Site	44	
CMH-Argentina		27% (12)
FC-Brazil		43% (19)
IHSS/HE-Honduras		2% (1)
INCMNSZ-Mexico		27% (12)
IMTAvH-Peru		0% (0)

Categorical variables are reported as percentages (count).

Continuous variables are reported as medians (interquartile range).

**Figure 1:**
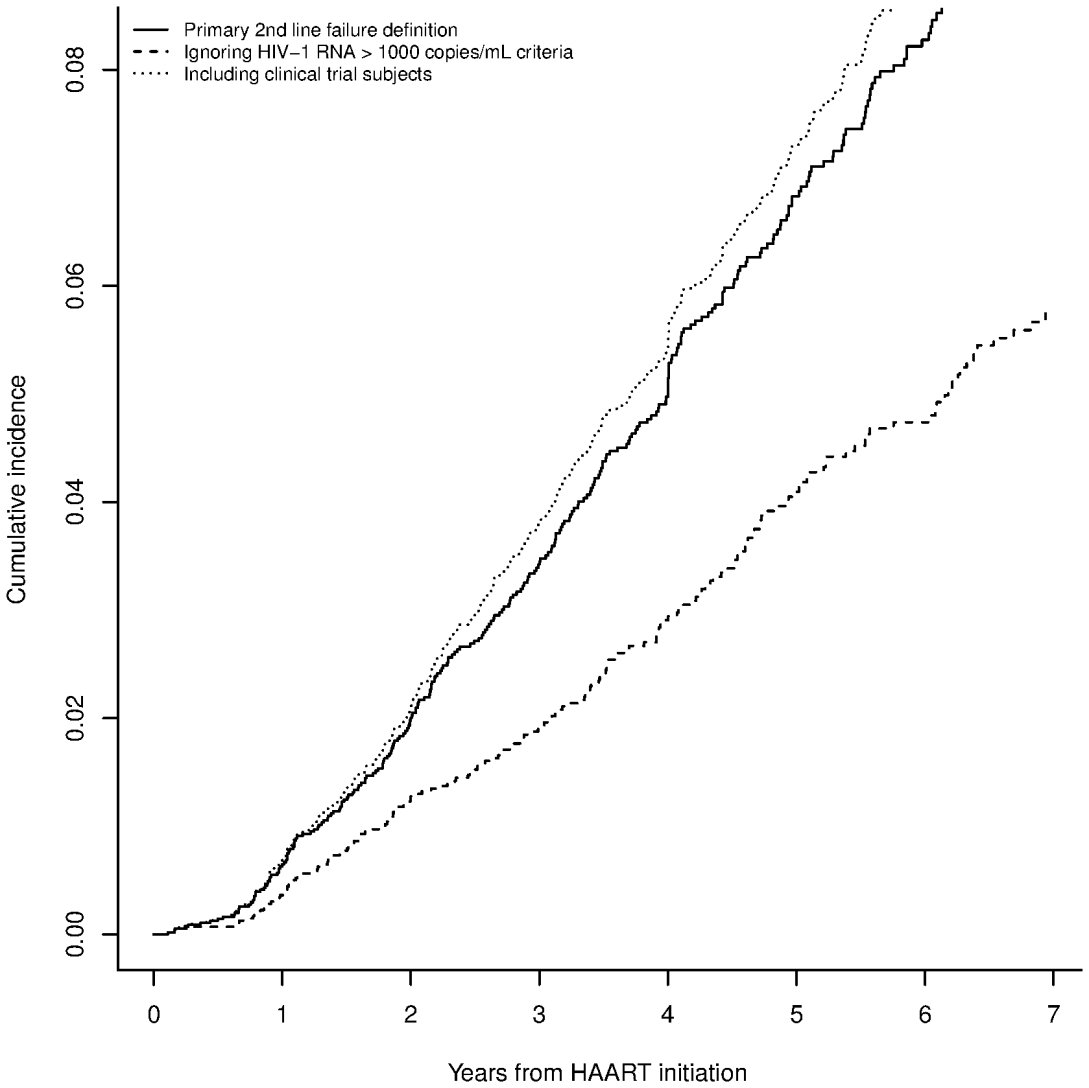
Cumulative incidence after HAART initiation of starting a third line regimen or failing a second line regimen.

As a crude measure for the need for third line therapy, we considered the composite endpoint, failure of a second line or start of a third line regimen. A total of 333 patients (5.7% of HAART initiators) experienced this composite event. The cumulative incidence of starting a third line or failing a second line regimen is shown in Figure 1. The estimated incidences at 3, 5, and 7 years after HAART initiation were 2.7% (95% confidence interval [CI] 2.2–3.2), 6.0%(95% CI 5.2–6.8), and 9.0% (95% CI 7.9–10.1), respectively. The dotted line in Figure 1 shows the estimated incidence using the above described more conservative definition of virologic failure.

Table 4 examines predictors at HAART initiation of subsequently failing a second line or starting a third line regimen. In multivariable analyses, female sex, younger age, and prior AIDS were associated with a higher hazard of experiencing the composite endpoint. Results were similar when HIV-1 RNA at first HAART initiation (missing for 35% of patients) was included in the analysis; HIV-1 RNA at HAART initiation was not a strong predictor of failing a second-line or starting a third-line regimen after adjusting for the other variables (p = 0.82).

**Table 4:** Hazard ratios for second line failure or third line regimen start.

	HR	95% CI	P
Male	0.65	(0.50, 0.85)	0.001
Age (years)			<0.001
20	2.76	(1.86, 4.10)	
30	1.52	(1.32, 1.75)	
40 (ref)	1.00		
50	0.85	(0.69, 1.05)	
AIDS at first HAART	2.17	(1.62, 2.90)	<0.001
Year of first HAART			0.37
2002	1.00	(0.77, 1.31)	
2004	0.99	(0.88, 1.11)	
2006 (ref)	1.00		
2008	1.08	(0.97, 1.21)	
2010	1.29	(0.90, 1.84)	
Probable route of infection			0.49
Heterosexual (ref)	1.00		
MSM	0.95	(0.70, 1.28)	
IDU	1.84	(0.80, 4.21)	
Other	0.97	(0.69, 1.37)	
CD4 at first HAART			0.07
50	1.32	(1.00, 1.75)	
100	1.20	(0.92, 1.58)	
200	1.09	(0.89, 1.33)	
350 (ref)	1.00		
Type of first HAART			0.79
NNRTI (ref)	1.00		
Other	0.96	(0.72, 1.28)	

In a sensitivity analysis, we repeated the primary analysis limited to the subset of 4988 patients whose first HAART regimen contained an NNRTI. Results remained largely consistent. Two hundred and forty-nine patients (5.0%) failed a second line regimen and 29 patients (0.6% of total) started a third line regimen. The estimated incidence of the composite endpoint was 2.8%, 5.6% and 8.5% at 3, 5, and 7 years. We also performed an additional sensitivity analysis including clinical trial participants (n = 635), excluding those who used third-line drugs as part of their initial regimen. The estimated incidence of the composite endpoint when including clinical trial participants was similar, albeit slightly higher, than the primary analysis: 3.0%, 6.4%, and 9.6% at 3, 5, and 7 years (dotted line in Figure 1). In another sensitivity analysis, we censored those who failed a second line regimen during a treatment interruption (10% of failures). The estimated incidence (3, 5, and 7 year estimates of 2.5%, 5.5%, and 8.1%, respectively) and risk factors were similar. In a final sensitivity analysis, we censored patients who had a gap between viral load measurements of more than a year; the estimated incidence was slightly lower: 2.7%, 5.2%, and 7.1% at 3, 5, and 7 years, respectively.

## Discussion

At five Latin American sites, we found very low rates of third line regimen initiation. Only 0.8% of HAART initiators started a third line regimen after a median of 3.5 years of follow-up. However, our data suggest that the need for third line regimens, assessed through use of third line or failure of second line, is much higher, with approximately 6% of patients needing a third line regimen within 5 years of starting antiretroviral therapy – five to ten times higher than the number who received one.

There are many possible reasons for the low rates of third line regimen use. Third line regimens are relatively new to Latin America and the cohort studied started first HAART after 2000. Limited drug availability and the high price of third line drugs are the most important barriers to implementing third line therapy in Latin America, and at a global level. Using the most updated international reference prices, a third line regimen consisting of ritonavir boosted darunavir, etravirine and raltegravir is 14 times more expensive than the WHO-recommended first line fixed dose combination [12]. In the region, low relatively market volume, weak generic competition, patent restrictions and tiered pricing policies continue maintaining prices higher than the international reference prices. The PAHO Strategic Fund, a revolving fund mechanism for international purchasing at reduced cost by taking advantage of savings offered by economies of scale, might represent an option for having access to antiretroviral third line drugs at lower price [13].

Patients participating in clinical trials were excluded from our analyses because of different relationships between clinical trial units and observational cohorts across CCASAnet sites. Some sites include data on clinical trial participants in their cohorts and others keep these data separately. In addition, many patients excluded were in trials that randomized antiretroviral-naïve patients to drugs considered in this study as components of third line regimens, but in their cases did not represent salvage drug use. However, it is possible that we underestimated the need for third line regimens because some patients may have chosen to enroll in clinical trials in order to access these drugs. In a sensitivity analysis including available data from patients who participated in clinical trials, the estimated use/need for third line regimens slightly increased. In addition, this study did not include patients who started therapy with non-HAART regimens, before 2000, or in other centers who may have had inadequate sequencing of regimens and were therefore at an increased risk of needing third line drugs. In addition, patients who may need third line drugs for reasons other than failure (such us toxicity or concomitant medication) were not accounted for.

We found that female sex, younger age, prior AIDS, and a lower CD4 count at baseline were associated with a higher hazard of experiencing the composite endpoint. This is the first time we have seen worse HAART outcomes among females in our cohort; in previous studies female sex was not associated with mortality, HAART change, or late presentation to care [14]–[16]. The higher risk of failure in females has been described by Caseiro et. al. in a study from Brazil where females had lower levels of education than men, although education was found not to be related to virologic failure [17]. The reason for the increased risk of virologic failure in women was unclear, and could have been due to differential rates of adherence or toxicity. A proportion of women may have taken antiretroviral drugs to prevent maternal-to-fetal transmission and then discontinued therapy after pregnancy. Younger age has been associated with failure in other studies, mainly due to lower adherence, treatment interruptions or depression [12], [18]–[21]. The observed association between advanced HIV disease at HAART initiation and the need for third line regimen is not unexpected, and provides further evidence of the need for early HIV diagnosis and treatment to improve outcomes.

This analysis has several limitations in addition to those already mentioned. Our measure of need for third line is a crude measure, which is certainly not completely accurate. It is a function of the frequency of viral load measurements in the database, highly variable between sites, and we do not have data on adherence. The former limitation may lead to under-estimation of need for third line regimens, the latter to unpredictable changes. Indeed, the site in Honduras had both the lowest estimated rate of the composite endpoint (data not shown) and the lowest frequency of viral load measurements. HIV/AIDS programs with limited access to virologic monitoring also tend to be those with limited access to third line regimens. Expansion of access to viral load monitoring should also prompt access to third line drugs for those in need. Adherence and drug resistance, well-established as determinants of treatment failure in other cohort studies [6], [7], have not been assessed in our cohort. Because we have not used HIV-1 genotyping data to characterize the population of patients initiating third line drugs or failing second line, we may have overestimated the need for third line therapy in our cohort. A small number of patients were known to have been off their antiretrovirals at the time of their second line failure. With that said, the rate of virologic failure while on the second regimen (31% of those starting a second line) was similar to estimates from other cohorts where rates of second line failure ranged from 8.6 to 37.3% [22].

Our sites by no means represent all of Latin America; we only have included a small number of sites in a portion of the countries in the region. Sites in CCASAnet are mainly referral centers in their respective countries and included patients may therefore have a different predisposition to requiring third-line drugs compared to those patients who do not participate in these cohorts. CCASAnet sites belong to different public health systems and follow local or national guidelines, which differ across countries. With only five sites we were not able to study the potential association between rates of failure and site-level variables, such as availability of food and transport subsidies and peer and other adherence support.

With the limitations noted, this study is the first to investigate need for third line regimens in Latin America and may be useful to policy makers for rough estimates of third line need [22]. Currently, third line regimens may be needed for up to 6% of patients after 5 years of ART. Despite much initial success in expanding access to ART, program enhancements such as options for third-line treatment are urgently needed to optimize health and survival among persons receiving ART in LAC. This study also highlights the need for early HIV diagnosis, effective and safe treatment options, and effective adherence strategies to reduce virologic failure and therefore the need for third-line drugs. In addition, greater access to routine virologic monitoring would allow detection of virologic failure in a timely manner.
